# Regulation of cardiomyocyte fate plasticity: a key strategy for cardiac regeneration

**DOI:** 10.1038/s41392-020-00413-2

**Published:** 2021-01-27

**Authors:** Rui Gong, Zuke Jiang, Naufal Zagidullin, Tianyi Liu, Benzhi Cai

**Affiliations:** 1 Department of Pharmacy at the Second Affiliated Hospital, and Department of Pharmacology (The Key Laboratory of Cardiovascular Research, Ministry of Education) at College of Pharmacy, Harbin Medical University, 150086 Harbin, China; 2 Department of Internal Diseases, Bashkir State Medical University, Ufa, 450008 Russia; 3 College of Pharmacy, University of Cincinnati, Cincinnati, OH USA

**Keywords:** Cardiology, Cardiovascular diseases

## Abstract

With the high morbidity and mortality rates, cardiovascular diseases have become one of the most concerning diseases worldwide. The heart of adult mammals can hardly regenerate naturally after injury because adult cardiomyocytes have already exited the cell cycle, which subseqently triggers cardiac remodeling and heart failure. Although a series of pharmacological treatments and surgical methods have been utilized to improve heart functions, they cannot replenish the massive loss of beating cardiomyocytes after injury. Here, we summarize the latest research progress in cardiac regeneration and heart repair through altering cardiomyocyte fate plasticity, which is emerging as an effective strategy to compensate for the loss of functional cardiomyocytes and improve the impaired heart functions. First, residual cardiomyocytes in damaged hearts re-enter the cell cycle to acquire the proliferative capacity by the modifications of cell cycle-related genes or regulation of growth-related signals. Additionally, non-cardiomyocytes such as cardiac fibroblasts, were shown to be reprogrammed into cardiomyocytes and thus favor the repair of damaged hearts. Moreover, pluripotent stem cells have been shown to transform into cardiomyocytes to promote heart healing after myocardial infarction (MI). Furthermore, in vitro and in vivo studies demonstrated that environmental oxygen, energy metabolism, extracellular factors, nerves, non-coding RNAs, etc. play the key regulatory functions in cardiac regeneration. These findings provide the theoretical basis of targeting cellular fate plasticity to induce cardiomyocyte proliferation or formation, and also provide the clues for stimulating heart repair after injury.

## Introduction

In the past few decades, the prevalence of cardiovascular diseases (CVDs) have increased significantly and become one of the leading causes of mortality worldwide.^1,2^ MI is the most common CVDs, accompanied by massive loss of cardiomyocytes and heart remodeling, which ultimately develops into heart failure and sudden cardiac death.^3,4^ Heart transplantation has been shown to be a feasible and effective method for severe MI and advanced heart failure.^5^ However, the poor availability of donated organs and some complications have limited its application. So, finding an effective way to replenish the loss of cardiomyocytes during heart attack has been a hot topic of interest.

Cardiomyocyte plasticity plays a critical role in cardiac adaptive responses such as myocardial remodeling and heart repair. In response to various stimuli, the heart will gradually gain appropriate renewal potential to replace necrotic or apoptotic cardiomyocytes after injury, bringing hope to patients with MI. Recently, increasing evidence has suggested that targeting the plasticity of cell fate is one new potential approach for cardiac regeneration, which can be mainly achieved by reprogramming non-cardiomyocytes into cardiomyocytes, the differentiation of pluripotent stem cells into cardiomyocytes, and the proliferation of pre-existing cardiomyocytes.

Cardiomyocytes account for 75% of left ventricular volume in healthy adults,^6^ and facilitate the blood pumping into the circulatory system by coordinating contraction and diastole. Mammalian cardiomyocytes have been thought as terminally differentiated cells that have little ability to proliferate. Recently, some groundbreaking studies has debunked this concept and provided the compelling evidence. Fetal and neonatal cardiomyocytes have been shown to proliferate and then repair the damaged tissues.^7^ Nevertheless, adult mammalian hearts hardly regenerate functional myocardium after injury due to inadequate cardiomyocyte renewal.^8,9^ However, recent studies have revealed that damaged hearts in MI are able to acquire regenerative potential by targeting some key signal pathways that induce cardiomyocytes to re-enter the cell cycle.

Non-cardiomyocytes are also important components of adult hearts, most of which are cardiac fibroblasts.^10^ It was lately indicated that cardiac fibroblasts can be directly reprogrammed into induced cardiac-like myocytes (iCLM) by introducing cardiogenic transcription factors, Gata4, Mef2c, and Tbx5 (GMT), thereby providing a potential source of cells for heart repair.^10–12^ It has also been demonstrated that regulation of some critical microRNAs (miRNAs) and epigenetic modification improve the efficiency of reprogramming non-cardiomyocytes into cardiomyocytes.^13–16^ Thus, reprogramming non-cardiomyocytes into cardiomyocytes in situ would represent a powerful and attractive alternative strategy for myocardial regeneration.

Pluripotent stem cell-based therapy has been suggested as a promising treatment for cardiovascular diseases.^17,18^ Pluripotent stem cells such as embryonic stem cells (ESCs) and induced pluripotent stem cells (iPSCs) can differentiate into almost any somatic cells including cardiac myocytes.^19^ Transplantation of pluripotent stem cell-derived cardiomyocytes combined with stem cells can not only significantly increase the retention of transplanted cardiomyocytes and improve the survival, but also promote neovascularization and anti-inflammatory through paracrine pathway, thereby repairing the damaged heart and improving cardiac functions.^20^

**Fig. 1:**
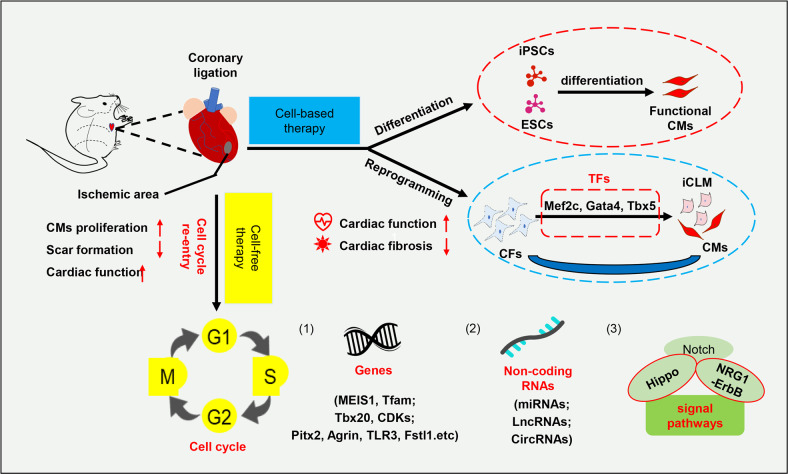
Schematic diagram of cell-based therapy and cell-free therapy for heart repair and cardiac function improvement after MI. In cell-based therapies, cell differentiation and trans-differentiation are introduced. Among them, iPSC-CMs and ESC-CMs obtained through in vitro differentiation were transplanted into the infarcted heart to survive and maintain stable cardiac implants, achieve iCLM function, and ultimately improve cardiac functions. Reprogramming large amounts of CFs, also present in the heart, into cardiomyocytes is also a heart repair strategy that can achieve orthotopic cardiomyocyte supplementation. In cell-free therapy, the main entry point is the re-entry of the cell cycle. Pre-existing cardiomyocytes re-enter the cell cycle to proliferate through intervention with cyclin modulators (such as cyclin or its dependent kinases (CDKs), and genetic modification with cycle arrest modulators (such as MEIS1) and cycle promoters (such as GATA4 and Tbx20), as well as other aspects of the microenvironment (for example, Pitx2, etc). In addition, some non-coding RNAs or various signal pathways promote or hinder heart repair and cardiac regeneration by regulating the expression of critical genes. CMs cardiomyocytes, CFs cardiac fibroblasts, CircRNAs circular RNAs, ESCs embryonic stem cells, Fstl1 follistatin-like 1, iCLM induced heart-like muscle cells, iPSCs induced pluripotent stem cells, LncRNAs long non-coding RNAs, miRNAs microRNAs, Tfam transcription factor A, mitochondrial, TLR3 Toll-like receptor 3, TFs transcription factors. ↑: upregulate; ↓: downregulate.

Up to now, a series of cell cycle regulators, signal pathways, non-coding RNAs, and other molecules have been shown to be involved in post-injury cardiomyocyte proliferation and heart repair, which may be used to develop innovative cardiac regenerative drugs for clinical application (Fig. 1).^21–26^ Besides, hypoxia, energy metabolism, extracellular matrix, nerve, epicardial factors, and inflammation and other factors, are also involved in the regulation of cardiomyocyte proliferation and heart repair. Targeting these factors in infarcted hearts become the new therapeutic strategy for cardiac regeneration. In this review, we summarize these progress on regulating the cell fate plasticity for cardiac regeneration and how these pathways could be targeted for therapeutic benefit.

## Reprogramming non-cardiomyocytes into cardiomyocytes

Generally, cardiac fibroblasts are activated after MI and recruited to the injured site to form scar tissue to replace the injured heart muscle. Therefore, reprogramming these cells into functional cardiomyocytes would be an ideal strategy for heart repair in response to ischemic injury. It was initially found that the transcription factor encoded by the myogenic regulator MYOD1 induces many types of cells to differentiate into skeletal muscles.^27^ Then, it was reported that after infected with transcription factors Oct3/4, Sox2, c-Myc, and Klf4 combined with retroviral transduction, fibroblasts can be reprogrammed into iPSCs.^28^ Interestingly, later studies found that in vitro fibroblasts also can be directly reprogrammed into iCLM after the integration of the transcription factors GMT, which provide a potential source of cells for heart repair.^11^ Previously, just a small portion of these cells were shown to be beating cardiomyocytes, so it has attracted much attention to improve the reprogramming efficiency.

Currently, endogenous mouse cardiac fibroblasts have been found converted into iCLM in vivo upon GMT or GHMT (GMT plus Hand2) transduction.^29–31^ GMT or GMT plus Mesp1 and Myocd combined with miR-133 are capable to regulate the gene expression of mouse embryonic fibroblasts (MEF), adult mouse cardiac fibroblasts (MCF) and human cardiac fibroblasts (HCF) from fibroblast phenotype to cardiomyocyte-like phenotype, and in turn accelerate cardiomyocytes reprogramming.^32^ Additionally, reprogramming efficiency of cardiomyocytes induced by GMT in vitro and in vivo was enhanced by transforming growth factors and Wnt inhibitors.^33^ Interestingly, epigenetic factors play the promoting or inhibitory roles in cardiac reprogramming.^34^ For example, inhibition of H3K4 methyltransferase Mll1 increased the efficiency of embryonic and cardiac fibroblasts transforming into functional iCLM.^35^ These findings provide new insights into molecular mechanisms of fibroblast-cardiomyocyte transformation. Single-cell transcriptomics have also been applied to reconstruct reprogramming trajectories. It provides a more comprehensive understanding of cellular reprogramming by identifying intermediate cell populations, signal pathways and potential regulators involved in iCLM reprogramming.^36^ Notably, direct reprogramming in vivo appears to improve post-MI heart function in mice more efficiently than in vitro, suggesting that the reprogramming effectiveness depends in part on functional cardiomyocytes, and other factors are also committed to heart repair together.^37^ Thus, although it has been revealed that targeting signal pathways, growth factors, miRNAs and small molecules further facilitate iCLM reprogramming, the more in-depth exploration of its regulatory mechanism is required before it is applied to the clinic.

Clustered, regularly interspaced short palindromic repeat (CRISPR)-Cas system is a prokaryotic immune system that confers resistance to foreign genetic elements.^38^ Because of its simplicity, versatility, specificity and high efficiency, it has been widely used in the biomedical field.^39,40^ The CRISPR/Cas9 system can directly activate the expression of downstream target genes with high precision.^41^ CRISPR interference was used to repress the expression of EGF-like domain 7 (Egfl7) in zebrafish to investigate its role in angiogenesis.^42^ Moreover, several studies showed that genetically engineered stem cells by CRISPR/Cas9 protected the damaged heart and improved cardiac functions.^43,44^ Currently, CRISPR/Cas9 can reprogram the human fibroblast lineage into induced cardiac progenitor cells, which then differentiate into cardiomyocytes, smooth muscle cells, and endothelial cells in vitro.^45^ The finding provides a new source of cells for disease modeling, drug screening, and rational and personalized cardiac cell therapy. Due to the off-target effect and unstable editing efficiency of gene editing, more work needs to be done in establishing effective and achievable delivery methods and identifying therapeutic targets.

## Differentiation of pluripotent stem cells into cardiomyocytes

Human pluripotent stem cells (hPSCs) are mainly consisted of human embryonic stem cells (hESCs) and human-induced pluripotent stem cells (hiPSCs), and have the capability to differentiate into almost any proliferative somatic cells.^19^ Previous studies suggested that hPSC-derived cardiomyocytes (hPSC-CMs) can be produced by simulating some developmental signaling cues.^46^ Indeed, hESCs are able to differentiate into functional cardiomyocytes following a mature cardiac differentiation protocol,^47–49^ and transplantation of hESCs-derived cardiomyocytes (hESC-CMs) and hiPSCs-derived cardiomyocytes (hiPSC-CMs) in vivo can promote the regeneration of the damaged heart and improve cardiac functions.^20,50^ Thus, hPSC-CMs have broad prospects in cardiac regeneration after injury. It has been reported that ESC-derived cardiomyocytes (ESC-CMs) express genes that play critical roles in cardiac development, and the electrophysiological and contractional phenotypes of the heart.^51,52^ Moreover, ESC-CMs transplanted into the infarcted heart can survive and improve cardiac functions.^53^ Nevertheless, transplantation of ESCs resulted in severe teratomas in the implanted area, which are attributed to the immune response and the accumulation of undifferentiated ESCs.^54–56^ IPSCs have unlimited proliferation capacity and can differentiate into cardiomyocytes.^46,57^ Cardiac systolic function in MI hearts was significantly improved at 4 and 12 weeks after transplantation of iPSC-derived cardiomyocytes (iPSC-CMs).^58^ However, as expected, the immaturity of iPSC-CMS in transplanted animals often leads to ventricular tachycardia. At the same time, iPSC-CMs trigger an immune response mediated by natural killer cells (NKCs), resulting in a low engrafting.^59^ Currently, attempts have been made to improve the implantation of pluripotent stem cell-based therapy and inhibit the occurrence of tumors and arrhythmias by obtaining high purity and mature iPSC-CMs (Table 1)^19,46,48,49,60,61^. Enrichment of cardiomyocytes by vascular cell adhesion molecule 1 (VCAMI)-coupled magnetic dynabeads^62^ and hydrogel^63^ or cell sheet holder^64^ have been shown to improve therapeutic effects. However, more investigations are required to determine the safety and effectiveness of pluripotent stem cell therapies, especially in clinical stage.

**Table 1:** Small molecules mediate the differentiation of human pluripotent stem cells into cardiomyocytes

Cell type	Small molecules	Description	Ref.
hiPSCs/hESCs	Gsk3 inhibitor CHIR99021, IWP2/IWP4	Activition of Wnt/catenin signal promotes hPSCs differentiation.	^46^
hESCs	SB203580 (a specific p38 MAP kinase inhibitor)	A specific p38 MAP kinase inhibitor can improve the efficiency of hESCs differentiation into CMs.	^48^
hESCs	PGI2, SB203580	Optimizing the concentration of PGI2 in serum-free medium or adding SB203580 can promote the differentiation of hESCs into CMs.	^49^
hiPSCs	Gsk3 inhibitor CHIR99021, Wnt-C59	Optimized chemically determined medium contributes to the production of high-purity cardiac troponin T (TNNT2)^+^ cells.	^60^
hiPSCs	XAV939 (a tankyrase inhibitor), IWP2 (a porcupine inhibitor)	Combination therapy of XAV939 and IWP2 results in higher efficiency in cardiac differentiation.	^61^

CMs cardiomyocytes, Gsk3 glycogen synthase kinase 3, *hPSCs* human pluripotent stem cells, *hESCs* human embryonic stem cells, *hiPSCs* human-induced pluripotent stem cells, *IWP2* inhibitor of Wnt production-2, *IWP4* inhibitor of Wnt production-4, PGI2 prostaglandin I2

It had been argued that cardiac stem cells (CSCs) could differentiate into cardiomyocytes, smooth muscle cells and endothelial cells,^65^ and exogenous implanted CSCs improved cardiac functions of damaged hearts.^66^ However, by utilizing advanced molecular and genetic techniques, lately reports uncovered that CSCs eventually do not transform into functional cardiomyocytes. The benefit of infarcted mice after intramyocardial injection of CSCs was shown to be associated with an acute inflammatory-based wound-healing response.^67,68^ It suggests that CSCs do not produce new cardiomyocytes and its effects on hearts after MI are complicated.

## Proliferation and mitosis of pre-existing cardiomyocytes

**Fig. 2:**
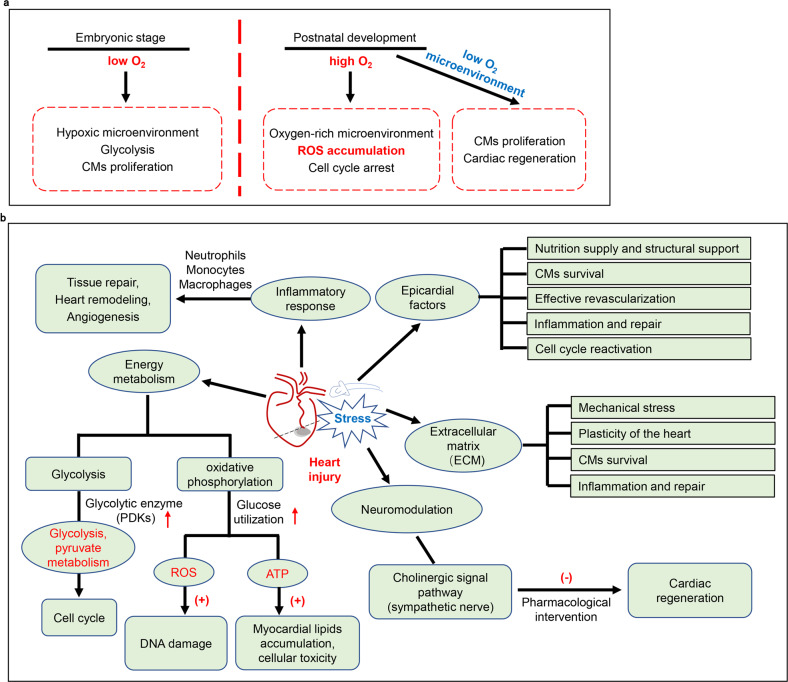
Physiological and pathological factors involved in the regulation of cardiomyocyte proliferation. **a** Comparison of embryonic development and post-natal cardiac microenvironment (oxygenated state) on cell proliferation potential. **b** Under stress or injury, the changes in pathological microenvironment play a vital role in heart repair and cardiac regeneration. CMs cardiomyocytes, ECM extracellular matrix, ROS reactive oxygen species. ↑: upregulate, (+): promote, (-): inhibit

**Fig. 3:**
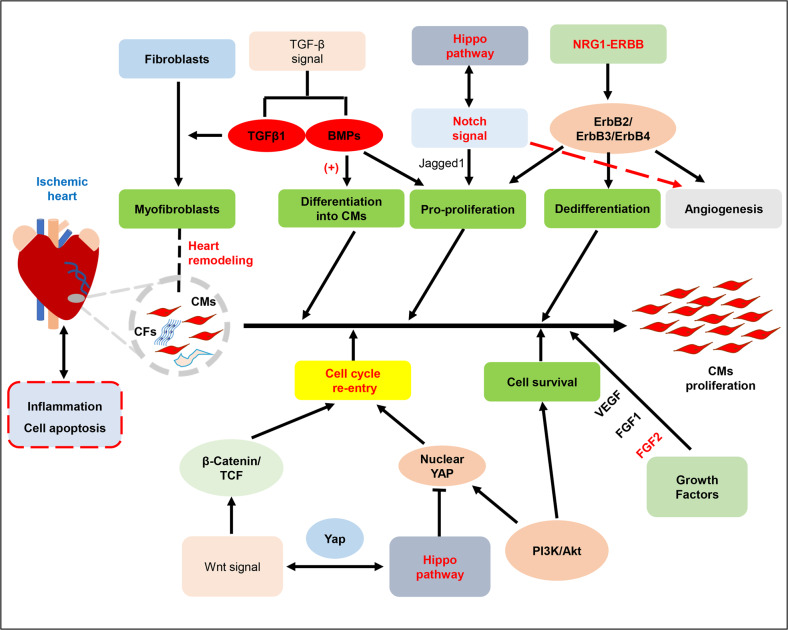
The main signal pathways involved in cardiac development and cardiac regeneration. Among them, Hippo, Notch, and NRG1-ERBB signaling pathways play a multi-directional coordination role in regulating heart repair and improving cardiac functions. Jagged 1 is a ligand for Notch receptor. Wnt and TGF-β pathways are indirectly involved in critical regulation of cardiomyocytes proliferation and damaged heart repair and remodeling. CMs cardiomyocytes, CFs cardiac fibroblasts, TGF-β transforming growth factor β. (+): promote

The endogenous cardiomyocytes of adult mice proliferate at the low level and cannot compensate for cardiomyocyte death caused by apoptosis and necrosis, so adult hearts have almost no reparative capacity after ischemic injury.^69^ Nevertheless, adult amphibians and zebrafish have a strong cardiac regeneration ability.^70–74^ Similarly, fetal and newborn mouse hearts can still regenerate myocardium through cell division after injury.^7,75^ Recently, increasing evidence showed that targeting the cell cycle of adult cardiomyocytes is able to induce pre-existing cardiomyocyte to proliferate, which has become one new strategy for cardiac regeneration after injury. The activation or inactivation of cardiomyocyte proliferation is linked to many pathophysiological factors and regulatory genes. Cardiac microenvironment, extracellular matrix, neuromodulation, etc have been found involved in cardiac regeneration by impacting cell proliferation (Fig. 2).^34,76–80^ Furthermore, some key regulatory genes have been found involved in cardiac regeneration by impacting different biological functions (such as cell proliferation and angiogenesis) (Fig. 3).^81–90^

### Pathophysiological factors in cardiac regeneration

#### Regulation of cardiac regeneration by oxidant stress

During heart development, microenvironment of the hearts changes from an anaerobic environment (hypoxia) to oxidative metabolism. The change leads to the production and accumulation of large amounts of reactive oxygen species (ROS), further destroying the genome.^77,78^ Elimination of ROS or inhibition of DNA damage response prolonged the maintenance period of post-natal cardiomyocyte proliferation potential, while hyperoxygenation and ROS donors shortened the regeneration maintenance period. These findings revealed a protective mechanism that mediates cardiomyocyte cycle arrest in exchange for oxygen-dependent aerobic metabolism. Additionally, when aerobic respiration of cardiomyocytes was suppressed in adult mice, it could reduce oxidative DNA damage and reactivate the mitosis of cardiomyocytes.^79^ These studies suggest that the pre-existing cardiomyocytes was able to re-enter cell cycle to proliferate with the change of heart microenvironment.

#### Energy metabolism mediates cardiac regeneration

As known, the energy supply of adult cardiomyocytes mainly comes from the oxidative phosphorylation of mitochondria.^78^ With the change of energy supply pattern before and after birth, the increase of oxidative stress plays a critical role in cell cycle arrest.^79,91^ Meanwhile, the inhibition of glycolysis resulted in impaired regeneration ability of neonatal mouse cardiomyocytes.^24,92^ Recent studies have revealed that pyruvate dehydrogenase kinase (PDK) regulates glycolysis and pyruvate metabolism of cardiomyocytes of the border area in zebrafish after injury.^93^ Moreover, the loss of PDK4 can increase the proliferation of cardiomyocytes and improve left ventricular function after MI and reduce remodeling.^94^ These findings suggest that energy metabolism plays a critical role in cardiomyocyte proliferation after injury. Additionally, mice overexpressing the nuclear receptor PPARβ/δ can increase the expression of type 4 glucose transporter (Glut4) in the myocardium to promote glucose utilization, and significantly reduce myocardial damage caused by I/R injury.^95^ Thus, the regulatory mechanism of energy metabolism beneficial to the repair and regeneration of the heart after injury is complicated and influenced by many factors.

#### Extracellular matrix (ECM) regulation of cardiac regeneration

The integrity of the ECM microenvironment plays critical roles in regulating cardiac systolic and diastolic function. Cardiac ECM not only provide mechanical support but also act as a key factor to regulate cell survival, growth and development. Under stress conditions, ECM macromolecules are involved in the pathogenesis of ventricular dysfunction and heart failure by driving a variety of cellular biological responses. It was reported that the ECM protein Agrin has a positive effect on the mitosis of cardiomyocytes derived from mouse and human-induced pluripotent stem cells.^23^. Increasing evidence suggest that ECM is a highly dynamic regulatory network in cardiac regeneration and plays a vital role under stress or in pathological conditions.^96^

#### The role of neuromodulation in cardiac regeneration

A large number of studies have shown that nerve signal plays critical roles in regulating cardiac regeneration.^97^ It has been shown that the nerves at the bottom of the limbs of amphibians cannot regenerate after being cut off before or after amputation.^98^ For instance, the development of arm morphology and the regeneration of other organisms, such as chicken embryos, suggesting that cholinergic neurons play a vital role in the reformation of biological structures.^99,100^ Newborn mice can regenerate the heart within one week of birth, while adult heart almost lose the ability to regenerate.^75^ The evidence showed that pharmacological interventions on cholinergic signaling transduction (sympathetic) are able to inhibit the development of the cell cycle after AR and MI in zebrafish and newborn mice, thus affecting cardiac regeneration.^80,101^ It provides important insights into the role of neuromodulation in cardiac regeneration.

#### The vital role of epicardial factors after injury

The epicardium is an important source of progenitor cells and provides nutrition and structural support for adult heart. Many studies have confirmed that the thin mesothelial cell layer covering the chamber-the epicardium is a key factor in the repair and regeneration of a damaged heart. The epicardium of the zebrafish heart is activated when it is damaged and is committed to cardiac regeneration by producing factors that maintain heart function.^102^ In mammalian heart repair, cardiomyocyte survival and blood vessel formation were supported by epicardial cells.^11,103^ Revascularization, which is critical to supporting tissue repair and heart functions after MI, is also regulated by epicardial and endocardial signalings.^104^ In addition, the increase of the expression and activity of follistatin-like 1 (Fstl1) in the epicardium can stimulate the re-entry of cell cycle, and ultimately improve the impaired cardiac functions and survival.^25^ Therefore, The epicardium also plays a crucial role in response to cardiac regeneration and angiogenesis after injury.

#### Inflammatory response and immune cells regulate heart repair

It has been known for decades that the immune system coordinates immune cells to regulate tissue repair, angiogenesis, and fibrosis. In the early stage of heart infarction, white blood cells (including neutrophils and monocytes) rapidly infiltrate the infarcted area, activating the production of myofibroblasts and vascular cells for cardiac repair.^105^ the subsets of macrophages with pro-inflammatory properties derived from Ly6C^high^ monocytes, which are recruited through chemokine dependent pathway, infiltrate into the infarcted area and maintained the pro-inflammatory environment.^106,107^ Studies have shown that macrophages are essential for the regeneration of the newborn heart.^108–110^ Macrophages infiltration induced by monocyte chemoattractant protein-1 (MCP-1) can improve cardiac functions in mice after MI.^111^ Therefore, an in-depth understanding of the behavior of immune cells involved in heart repair will help develop new therapies for MI.

### Cell cycle regulators

The intervention of cell cycle regulators, such as cyclin or its dependent kinases (CDKs) enables the remaining cardiomyocytes to re-enter the cell cycle and maintain the proliferation signaling.^26,112^ It has been shown that cyclin mutant kinase 9 (CDK9) is involved in an important signal pathway for cardiac hypertrophy.^113^ CDK9 also plays a key role in responding to myocardial injury by directly binding to GATA-binding protein 4 (GATA4).^114^ This association provides theoretical support for CDK9 to paly a regulatory role in cardiomyocyte proliferation.^115^ It has been shown that CDK9 ablation inhibit the proliferation of zebrafish cardiomyocytes and has a negative effect on the repair of laser-damaged hearts.^116^ Later, it was found that endogenous CDK inhibitors trigger adult cardiomyocytes to re-enter the cell cycle and proliferate actively.^117^ These studies indicate that CDKs play a key role in regulating cardiomyocyte proliferation.

The myeloid ecotropic viral integration site 1 homolog (MEIS1), is a member of the TALE homeobox gene family, is a regulator of cell cycle arrest in post-natal cardiomyocytes. It was uncovered that loss of MEIS1 in adult mice induced cardiomyocytes to re-enter the cell cycle.^7^ Moreover, the synergistic effect of MEIS1 and its cofactor homeobox B13 (Hoxb13) can jointly regulate the proliferation window of cardiomyocytes after birth and the repair and regeneration of the adult heart.^118^ On the contrary, Tbx20, a member of the Tbx1 subfamily of T-box (Tbx) genes, directly repressed the expression of cell cycle inhibitors p21, MEIS1 and Btg2. Overexpression of Tbx20 in adult mouse cardiomyocytes can promote cell proliferation and significantly improve heart repair after MI.^119–121^ Besides, Tbx20 participated in the signaling cascade of Tbx20-PROK2-PROKR1 and regulated angiogenesis.^122^ The above findings provide further mechanistic insights into the link between cardiomyocyte proliferation and cardiac regeneration.

### Signal pathways in cardiac regeneration

#### Hippo pathway

Recent studies have suggested that Hippo pathway plays a pivotal role in regulating cardiomyocytes proliferation and can affect the heart size through regulation of apoptosis, proliferation, and cellular fate.^123–125^ Hippo pathway signaling cascade is highly conserved in mammals. Its core components are MST1 or MST2, LATS1 or LATS2 kinases and two accessory molecules SAV1 and MOB kinase activator 1 (MOB1).^126^ Activation of upstream components of the Hippo pathway, including Mst1 and Lats2, phosphorylates Yes-associated protein (Yap), which is a transcription factor that shuttle between the cytoplasm and nucleus to participates in cell survival and proliferation.^127^ The decrease of Yap binding to TEAD1, a transcription enhancer in the nucleus, causes a decrease in the expression of genes involved in cell growth and survival.^128^ Conversely, the inactivation of the Hippo pathway or activation of its downstream effector, the Yap transcription co-activator, promotes cardiac regeneration and improves heart function.^70,129–132^ Overexpression of activated Yap is sufficient to promote mitosis of cardiomyocytes. Additionally, some evidence suggests that Hippo signaling can regulate the cell cycle re-entry of adult cardiomyocytes. The genetic depletion interferes with the Hippo pathway to stimulate cardiomyocytes through the S phase, mitosis, and cytokinesis, exhibiting greater re-entry of the cardiomyocyte cell cycle.^133^ Thus, targeting Hippo pathway to regulate cardiomyocytes fate may be an attractive approach for heart repair after MI. However, the inhibited Hippo pathway can also activate a positive feedback mechanism called Yap-TEAD1-Oncostatin M (OSM), which eventually worsens cardiac dysfunction caused by pressure overload.^131^

#### Notch signaling pathway

The repair and regeneration of organs usually involves response to cellular signal pathways ralated to its development. Many studies in mice and zebrafish have confirmed that Notch signal plays a vital role in regulating heart repair and cardiac regeneration.^134^ Notch signal was found to be activated after heart injury in zebrafish, with the increased expression of various Notch receptors and ligands.^135^ The overall inhibition of the Notch signaling hindered cardiomyocyte proliferation and resulted in scarring.^136,137^ Inhibition of Notch signal in neonatal cardiomyocytes blocked cell proliferation and induced cell apoptosis, suggesting that Notch signal may be manipulated to affect the division of postnatal cardiomyocytes.^85^ In the damaged adult hearts, only the endocardial and epicardial cells near the infracted region expressed the Notch-related genes, suggesting that activation of the Notch pathway occurs in the endocardium and the effect of Notch signaling activation on myocardium may be indirect.^138^ Additionally, ectopic activation of Notch signaling in myocardium limits the pathological remodeling process of the heart after MI.^139^ Moreover, the regulation of cardiac development is regulated by a complex network of multiple signal pathways, such as Notch signal, Hippo pathway and so on.

#### Neuregulin1 (NRG1) -ErbB4 signaling

The NRG1 gene is a member of the epidermal growth factor (EGF) gene family and exerts biological functions through the ErbB family of tyrosine kinase receptors, including ErbB1, 2, 3, and 4.^140,141^ The myocardium of germline mice deficient in the NRG1, ErbB2, or ErbB4 genes thinned. These genes are necessary during the second trimester to produce fetal cardiomyocytes.^142–144^ In zebrafish heart, blocking NRG1 signal inhibited injury-induced cardiomyocyte proliferation. In the absence of heart damage, the reactivation of NRG1 may stimulate many signs of cardiac regeneration.^81^ These results suggest that NRG1 is the key node between myocardial injury and physiological cardiac regeneration. Additionally, activation of NRG1/ErbB4 signaling pathways induced the proliferation of differentiated cardiomyocytes, thereby enhancing cardiac regeneration after myocardial injury and improving heart functions. Importantly, NRG1-induced myocardial regeneration may attenuate the hypertrophic phenotype after MI, thus improving ventricular remodeling.^145,146^ This study presents one possible way to repair the heart by delivering recombinant growth factors. Additionally, on the basis of the fact that NRG1 stimulated cardiomyocyte proliferation in young adult mice,^145,147,148^ studies have found that NRG1 can also induce cardiomyocyte proliferation in infants under 6 months of age with heart disease.^149^ It suggests that NRG1 signal pathway is conservative and may serve as an effective therapeutic strategy for children with heart diseases.

### Non-coding RNAs

**Fig. 4:**
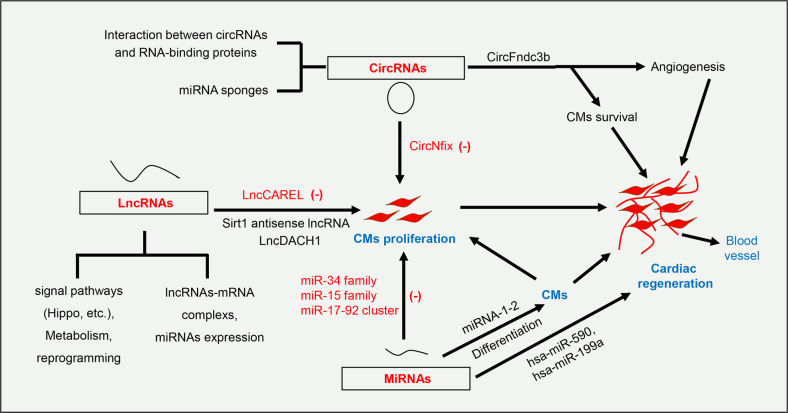
The vital regulatory roles of non-coding RNAs in cardiac regeneration after injury. MiRNAs regulate the critical process of heart repair and subsequent remodeling after infarction. Among them, miRNA (miR-34 family, miR-15 family, and miR-17-92 cluster), lncRNA (LncCAREL) and circRNA (CircNfix) negatively regulate the proliferation of cardiomyocytes. CircRNAs also play a critical role in controlling cardiomyocyte proliferation, angiogenesis and even tissue regeneration. CMs cardiomyocytes, CircRNA circular RNA, LncRNA long non-coding RNA, miRNAs microRNAs. (-): inhibit

Non-coding RNAs such as small non-coding RNAs (miRNAs) and long non-coding RNAs (lncRNAs),^150^ are functional RNA molecules that almost do not encode proteins.^151^ Recently, protective functions of some miRNAs and lncRNAs in cardiac regeneration and heart repair have been verified.^152,153^ In addition, circular RNA (circRNA) forms a covalently closed loop, which help to maintain its stability and enhance its miRNA/protein binding ability.^154^ There is evidence that certain types of circRNAs may play a significant role in controlling cell proliferation, development and even tissue regeneration (Fig. 4).^155,156^

#### MicroRNAs (miRNAs)

MiRNAs play a critical role in gene expression by binding to the 3’UTR of target mRNA. MiRNA binding can induce translational blocking of mRNA or direct mRNA cleavage.^157^ MiRNAs regulate the growth and development of the heart.^158,159^ Recent studies have demonstrated that the exogenous administration of miRNAs such as hsa-miR-590 and hsa-miR-199a stimulated cell cycle re-ertry of isolated cardiomyocytes, and promoted the regeneration of mouse hearts after MI.^160–162^ Some miRNAs also showed the capability to negatively regulate cardiomyocytes proliferation and cell cycle re-entry.^163,164^ For instance, MI can induce the expression of pro-apoptotic miR-34 family members. The inhibited expression of miR-34 family members or by increasing the expression of target genes (such as Vinculin, Pofut1and PNUTS) can effectively improve the impaired cardiac functions.^165,166^ It has been found that miRNAs can regulate a variety of signaling pathways involved in cardiac regeneration,^167,168^ including the MAPK signal, Hippo signal,^169^ Wnt signal, PI3K-Akt signal, and pluripotency pathways. Interestingly, miRNAs have also been shown to regulate the differentiation of stem cells into cardiomyocytes.^170^

#### LncRNAs

A large body of evidence has showed that lncRNA plays a unique role in cardiovascular diseases.^171–173^ Regulation of cell cycle genes is a feasible way to induce cardiomyocyte cycle re-entry.^7^ Studies have shown that lncRNAs can play a crucial function in cardiac regeneration by promoting the proliferation of pre-existing cardiomyocytes. Although lncRNAs do not translate into proteins, they have been shown to regulate the cell cycle re-entry of cardiomyocytes.^174,175^ Mechanistic analysis revealed that lncRNAs are also involved in multiple signal and metabolic pathways related to cardiac regeneration, such as Hippo pathway.^176^ Further, lncRNA can interact with the 3’UTR of mRNA to form molecular complexes to improve the stability of the mRNA and ultimately produce beneficial biological functions in heart failure.^177^ Therefore, it is necessary to further explore the regulatory mechanisms of some key lncRNAs on cardiomyocyte proliferation.

#### CircRNAs

In recent years, circRNAs have been recognized as a new type of non-coding RNAs with regulatory functions. It has a typical covalently closed structure, highly stable expression, and resistance to nucleases.^178^ CircRNA microarray analysis uncovered that there were significant differences in the expression of some circRNAs in newborn and adult mouse hearts, suggesting that circRNAs may be involved in heart development. Generally, circRNAs can interact with RNA-binding proteins to form complexes that promote the expression of key downstream targets to exert their biological effects. For example, circFndc3b can interact with RNA-binding proteins to promote the expression of VEGF, thereby promoting the reduction of cell apoptosis and improving cardiac regeneration.^179^ Besides, circRNA, as a miRNA sponge, regulate the expression of its downstream molecules, ultimately activating or inactivating relevant signaling pathways and generating corresponding biological functions.^180^ It was found that loss of circNfix promoted cardiomyocyte proliferation. Mechanically, On the one hand, circNfix as a sponge of miR-214 promoted GSK-3 expression and further inhibited the activity of catenin. Besides, circNfix-related super-enhancers regulated the expression of circNfix by recruiting the transcription factor MEIS1 and eventually served as an effective therapeutic target for improving heart function and preventing heart failure after MI.^181^

Non-coding RNAs are used as a potential target for the treatment of cardiovascular diseases, but needs further evaluation in pre-clinical studies and clinical trials. The exploration of the potential regulatory mechanisms of non-coding RNAs in various pathological processes will favor the deeper understanding of cardiac homeostasis and the development of cardiovascular diseases. In the future, non-coding RNAs will become an important part of the emerging field of cardiac regenerative medicine.

## Conclusion

Collectively, targeting the cell fate plasticity is emerging as a promising and essential strategy for repairing damaged hearts. Some important approaches for cardiac regeneration have been summarized and described here: Promoting cell cycle re-entry and mitosis of pre-existing cardiomyocytes in damaged hearts; Reprogramming non-cardiomyocytes such as cardiac fibroblasts into cardiomyocytes; Inducing pluripotent stem cells such as ESCs and iPSCs differentiate into cardiomyocytes. Cardiacogenesis and cardiomyocyte proliferation are regulated by cell cycle regulators, non-coding RNAs, etc, which are also associated with multiple signaling pathways, actually forming a highly coordinated and orderly network. All the findings provide new insights into underlying mechanisms of cardiac development and regeneration, and also confer novel therapeutic approaches for heart damage. In the future, multidisciplinary cooperation and in-depth understanding of cardiomyocyte fate plasticity are of great significance for guiding the treatment of cardiovascular diseases. Of course, the approaches to induce cardiac regeneration are beyond the three strategies we mentioned above. We need continue to focus on the treatment theories and technologies in this field.

## Challenges and future prospects

Cardiomyocyte differentiation of pluripotent stem cells is an important way to induce cardiac regeneration, but some moral disputes may arise due to the particularity of its source. At present, their clinical safety and efficacy have yet to be verified in clinical trials. Additionally, the occurrence of arrhythmias and severe immune response after transplantation in vivo also indicated that cardiomyocytes derived from pluripotent stem cells are immature and lack the function of adult cardiomyocytes. In recent years, some progress has been made in the study of signaling pathways regulating the proliferation of cardiomyocytes, but the effects of these pathways still need to be further verified in animal models closer to humans, so as to evaluate their possible therapeutic effects and side effects in a more detailed and comprehensive manner. In addition, non-coding RNAs have been identified as a vital new regulator of cardiovascular risk, and can be used as a diagnostic biomarker for CVDs.^182^ But its potential as a clinical therapeutic drug remains to be evaluated in a large number of clinical trials. The proliferative ability of cardiomyocytes also involves different pathophysiological microenvironment, including inflammation, ROS, extracellular matrix, neuromodulation, etc. These pathophysiological microenvironments enrich our understanding about cardiac regeneration events. However, their mechanisms in heart repair after MI needs to be further explored in a more comprehensive way. The reprogramming of fibroblasts into iCLM is able to repair infarcted myocardium and improve cardiac functions. In the future, more work should be committed to exploring the key regulators and enhancing the efficiency of non-cardiomyocyte reprogramming.
